# Thymoma with suspected secondary myocardial infarction in a red panda

**DOI:** 10.1177/10406387261422673

**Published:** 2026-04-04

**Authors:** Susanne Rau, Andreas Bernhard, Regina Scheller, Jan Schinköthe

**Affiliations:** Institute of Veterinary Pathology, Faculty of Veterinary Medicine, Leipzig University, Leipzig, Germany; Zoo Leipzig, Leipzig, Germany; Saxon State Laboratory of Health and Veterinary Affairs, Leipzig, Germany; Institute of Veterinary Pathology, Faculty of Veterinary Medicine, Leipzig University, Leipzig, Germany

**Keywords:** *Ailuridae*, MINOCA, myocardial infarction, silicosis, thymoma, x-ray emission

## Abstract

A 17-y-old, female red panda (*Ailurus fulgens*) with unusual restlessness and a poor prognosis was euthanized and submitted for autopsy. Postmortem examination revealed a large cystic mass within the cranial mediastinum that severely compressed the lung. Histologic analysis classified the mass as a thymoma with abundant small lymphocytes and recognizable type B2 epithelial cells. Additionally, the heart had several chronic myocardial infarcts within the left ventricular free wall and septum. Granulomatous lymphadenitis and prominent anthracosilicosis were noted in the tracheobronchial lymph nodes. Secondary findings included interstitial nephritis, splenic myelolipoma, dental attrition, endocardiosis, and ulcerative gastritis. Expansile thymomas are known to cause myocardial infarction with nonobstructive coronary arteries. We propose a similar pathogenesis in this case.

A 17-y-old, female red panda (*Ailurus fulgens*) was suddenly and unusually restless and hyporectic. The animal was housed in an outdoor enclosure (275 m^2^) bordered by a concrete wall with artificial rock cladding, a barbed wire fence, and glass panels. Additionally, the panda had access to indoor boxes (15 m^2^) made of concrete with a floor coated with epoxy resin. Five days after the first appearance of clinical signs, the animal was found in a non-responsive condition with myasis of the ano-cutaneous region and hindlimbs. Pre-terminal blood analysis revealed elevated concentrations of creatinine (435 µmol/L; RI: 35–159 µmol/L), urea nitrogen (62 mmol/L; RI: 4–20 mmol/L), and phosphate (5.1 mmol/L; RI: 0.6–2.9 mmol/L) compared with physiologic values of the red panda.^18^ Given the overall clinical impression and poor prognosis, the animal was euthanized, and the carcass was submitted for postmortem examination to the Institute for Veterinary Pathology (Leipzig University, Leipzig, Germany). During autopsy, samples from the brain, heart, lungs, spleen, liver, and kidneys, as well as the trachea, tracheobronchial lymph nodes, thyroid glands, stomach, small and large intestines, jejunal lymph node, pancreas, ovaries, teeth, nasal cavity, and a mediastinal mass were collected for histologic examination.

In the thoracic cavity, a 7.0 × 5.5 × 3.0-cm cystic mass was present within the cranial mediastinum (**Fig. 1A**), near the heart. It contained ~70 mL of clear orange fluid and caused atelectasis of the adjacent lung. The mass had an ~2-mm-thick wall, with several smaller solid nodules up to 1.5 × 1.5 × 0.5 cm (Fig. 1A). Histologically, the solid wall-like parts of the cystic mass consisted of numerous thymic epithelial cells (**Fig. 1B**) arranged in frequently merging clusters. Neoplastic epithelial cells featured bean-shaped nuclei with an open chromatin structure, dense peripheral chromatin condensation, and abundant pale eosinophilic cytoplasm (**Fig. 1C**), along with moderate anisocytosis and anisokaryosis. The mitotic count was low (1 mitotic figure/2.37 mm^2^, scope FN22). Single neoplastic cells also infiltrated the adjacent mediastinal fatty tissue in a few areas. In addition, many small lymphocytes without signs of atypia and single thymic corpuscles were present (Fig. 1C, inset). To delineate individual cell populations, immunohistochemistry (IHC) using selected cell markers was performed (**Table 1**). The neoplastic thymic epithelial cells had strong pan-cytokeratin immunolabeling (**Fig. 1D**) and a sheet-like growth pattern, which was unexpected compared with the H&E-stained sections. Moreover, moderate numbers of CD3-positive T lymphocytes were observed and were interspersed among or formed discrete nodules that separated the neoplastic epithelial cells (**Fig. 1E**). Discrete foci of CD20-positive B lymphocytes were also seen (**Fig. 1F**).

**Figure 1.**
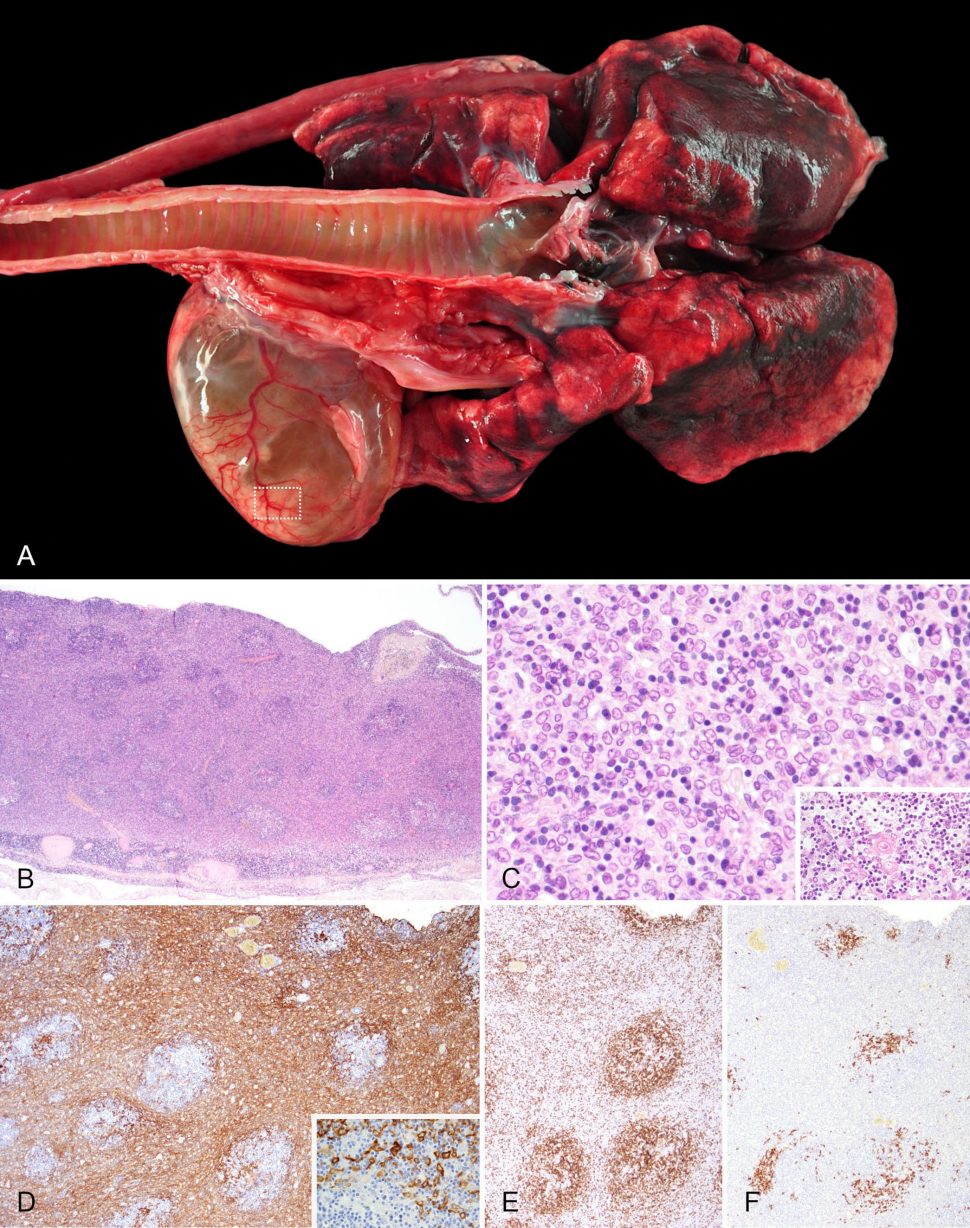
Cranial mediastinal thymoma in a red panda. **A.** A large, partially well-vascularized, opaque-to-translucent cystic mass cranial to the lung. Moderate pulmonary congestion, mild emphysema, and numerous pinpoint-to-confluent areas of anthracosis. **B.** The cyst wall (dashed rectangle in Fig. 1A) of an expansile, epithelial neoplasm has moderate numbers of small, non-neoplastic lymphocytes, partly aggregated in follicles. H&E. **C.** Numerous polygonal neoplastic epithelial cells with indistinct cell borders are arranged in frequently merging clusters infiltrated by moderate numbers of small, regular lymphocytes. Inset: a thymic corpuscle. H&E. **D.** Tumor cells are strongly positive for pan-cytokeratin. Inset: detail of pan-cytokeratin–positive tumor cells. AE1/AE immunohistochemistry (IHC). **E.** Moderate numbers of individualized or follicle-like aggregated CD3+ T cells are present. IHC. **F.** A few CD20+ B cells are observed, often in clusters; the spaces that labeled as CD3+ T cells in Fig. 1E are largely devoid of CD20+ B cells. IHC.

**Table 1.** Antibodies used for immunohistochemistry of a thymoma in a red panda.

Primary antibody	Target antigen	Host	Source	Clone	Dilution	Antigen retrieval method	Detection system
Pan-cytokeratin	AE1/AE3	Mouse	Dako (M3515)	AE1/AE3	1:50	HIER citrate buffer, pH 6	Vectastain Elite ABC kit; 1:200 (Vector, BA-9200)
CD3	CD3-complex, T3	Rabbit	Dako (A0452)		1:500	HIER citrate buffer, pH 6	Vectastain Elite ABC kit; 1:200 (Vector, BA-1000)
CD20	CD20	Rabbit	ThermoFisher (PA5-16701)		1:500	HIER citrate buffer, pH 6	Vectastain Elite ABC kit; 1:200 (Vector, BA-1000)

HIER = heat-induced epitope retrieval. Diaminobenzidine was the chromogen in all instances.

Furthermore, lesions were present in the left ventricular free wall and the adjacent septum. Approximately halfway from the heart base in the subatrial papillary muscle, the myocardium along the course of the paraconal interventricular branch of the left coronary artery had a thinned 1-cm area (**Fig. 2A**). At this site, the transversely sectioned myocardial wall was no more than 2 mm thick and had a slight outward protrusion (**Fig. 2B**), interpreted as a myocardial aneurysm. A similar 0.8-cm lesion was found in the subauricular papillary muscle, as well as a 0.3-cm lesion in the septum. Histologically, the described lesions had marked loss of cardiomyocytes with replacement fibrosis and minor fatty degeneration (**Fig. 2C**). Even after intense transversal 1-mm step-sectioning of the myocardium along the paraconal interventricular branch, we did not find evidence of intraluminal thrombi. Picrosirius red–stained slides highlighted extensive replacement fibrosis and revealed that only minor remnants of cardiomyocytes remained (**Fig. 2D**). The overall macroscopic and microscopic findings allowed the interpretation of 3 distinct chronic myocardial infarcts, one of which formed a myocardial aneurysm. Lesions were not evident in the remainder of the cardiac tissue examined.

**Figure 2.**
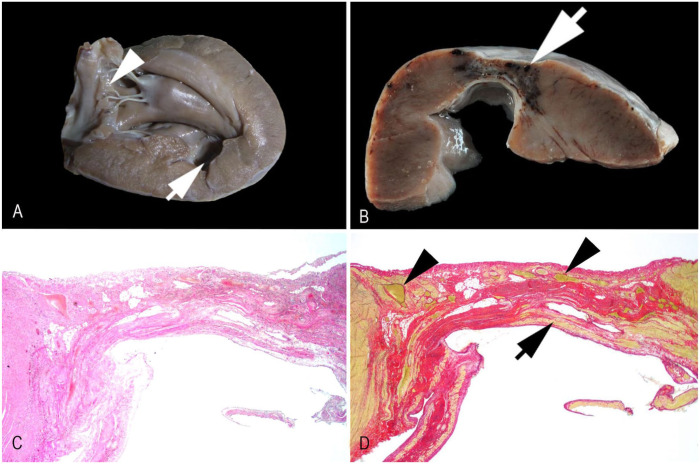
Myocardial infarcts in the left ventricle and septum of a red panda. **A.** Focal thinning of the septal myocardium (arrow). Mild, multifocal myxomatous degeneration of the mitral valve (arrowhead). **B.** Aneurysmal fibrosis and multiple congested vessels in the adjacent left ventricular free wall (arrow). **C.** Prominent replacement fibrosis and mild-to-moderate amounts of fibrofatty tissue within the aneurysmal region. H&E. **D.** Collagen fibers (red), with only remnants of yellow-staining myocardium (arrow) and numerous dilated, congested blood vessels (arrowheads). Picrosirius red stain.

Additionally, the tracheobronchial lymph nodes were marked by severe granulomatous lymphadenitis with caseating and non-caseating granulomas ( **Suppl. Fig. 1A–C** ). Both culture and IHC (rabbit polyclonal anti-purified protein derivative of *Mycobacterium tuberculosis*, OBT0947; Bio-Rad) of lymph node tissue for mycobacteria yielded negative results. Instead, using high-power microscopy and polarized light, we detected— in addition to carbon particles—birefringent amorphous crystals (up to 15 × 3 µm) in areas of caseous necrosis and within the cytoplasm of numerous macrophages ( **Suppl. Fig. 1D, 1E** ). We then performed scanning electron microscopy (SEM; EVO LS 15, Zeiss) on a deparaffinized, consecutive, formalin-fixed, paraffin-embedded section of the focus of granulomatous lymphadenitis with crystals (**Suppl. Fig. 1F**). Subsequently, energy-dispersive x-ray spectroscopy mapping (VPSE-G4, Smart EDX detector; Zeiss) revealed abundant silica-specific signals in macrophages, which colocalized with aluminum-specific signals ( **Suppl. Fig. 1G, 1H** ). Overall, in energy-dispersive x-ray (EDX) mapping of the analyzed area (222 × 172 µm), the relative abundance of elements by weight was: oxygen (59.0%), silicon (16.9%), phosphorus (5.3%), aluminum (4.0%), sodium (3.9%), and calcium (2.9%). Macrophages containing carbon particles, as well as peribronchiolar macrophages with the same intracytoplasmic crystals observed in the lymph nodes, were also present in the lung (data not shown), which grossly had mild-to-moderate anthracosis.

Bacteriologic and mycologic analyses of lung, kidneys, spleen, liver, small and large intestine, and tracheobronchial lymph nodes revealed mild-to-moderate numbers of *Salmonella* spp., as well as a mixed bacterial flora, including *Clostridium perfringens*, *Pseudomonas* spp., *Escherichia coli*, *Enterococcus* spp., *Streptococcus* spp., *Aspergillus* spp., and *Fusarium* spp. Furthermore, PCR testing of lung tissue was positive for *Mycoplasma* spp.

Additional findings included moderate, confluent bilateral renal cortical atrophy with interstitial lymphohistioplasmacytic nephritis and fibrosis, a splenic myelolipoma, and prominent dental attrition with parodontitis and gingivitis. Mild myxoid degeneration of the mitral valve (Fig. 2A) and mild ulcerative gastritis were also noted.

Thymoma is a neoplasm of predominantly middle-aged to geriatric individuals without any known sex predisposition. The neoplasm occurs in the cranial mediastinum and is found in a multitude of domestic animals with variable frequency. Middle-aged female goats are particularly prone to develop thymomas, but dogs, cats, sheep,^15^ and domestic rabbits^11^ are also affected infrequently. Thymomas are reported in wildlife species as well^9^; however, a thorough search in Google, PubMed, CAB Direct, Web of Science, and Scopus revealed no cases in the red panda, suggesting that descriptions of this neoplasm have not yet been peer-reviewed and published for this species.

Thymomas often grow cystically and usually have benign biological behavior, but can be locally invasive, as in our case. Their histologic appearance is heterogeneous, comprised mainly of neoplastic epithelial cells and variable numbers of infiltrating lymphocytes. Various subtypes can be differentiated based on the WHO classification of thymic epithelial neoplasms for humans.^7^ This classification is also commonly applied to thymic epithelial neoplasms in veterinary species, although data analyzing the correlation between histologic subtypes and clinical outcomes in animals other than the dog are insufficient.^9,11,14^ Following the WHO criteria, the overall histologic and IHC findings in our case are consistent with a B2 thymoma, featuring clusters of pan-cytokeratin–positive neoplastic thymic epithelial cells intermingled with moderate numbers of CD3-positive T lymphocytes that form prominent follicular structures.

Although some tumor cells infiltrated the adjacent mediastinal tissue, which could suggest a thymic carcinoma rather than a thymoma, we ruled out this diagnosis because the infiltrates involved only small numbers of neoplastic cells in few areas and, more importantly, all other histologic findings were consistent with a benign neoplasm (e.g., low mitotic count and absence of malignant cellular features). Also, the pan-cytokeratin–stained slide gave the impression of a prominent sheet-like growth of the thymic epithelial cells, leading to a B3 designation. However, given that classification relies on H&E-stained tissues, and multiple follicular-like structures along with moderate numbers of lymphocytes were noted, we classified the neoplasm as a B2 thymoma.

Clinical signs accompanying thymomas can vary widely. These include no signs at all and those resulting mostly from compression of the adjacent tissue (which causes respiratory or cardiovascular distress), to a number of paraneoplastic syndromes,^15,16^ such as myasthenia gravis, exfoliative dermatitis, hypercalcemia, thrombocytopenia, anemia, and granulocytopenia.^15^ Given that our case not only suffered from a B2 thymoma but also from several myocardial infarcts along the course of the paraconal interventricular branch, we propose that these 2 findings might be etiologically linked.

The overall morphologic and histopathologic characteristics of the observed myocardial infarcts were consistent with chronic lesions; hence, we assume that the causative event occurred at least several weeks to months prior. Not only is myocardial aneurysm known to be a frequent long-term effect of myocardial infarction in humans,^8^ but cardiovascular disease is also described as the leading cause of death in geriatric red pandas, with myocardial fibrosis being a common feature.^4^ Unfortunately, the exact anatomic location and extent of these lesions are usually not documented, except in one case report that also revealed fibrous changes in the myocardium of the left ventricular free wall and septum,^5^ similar to our report.

Given the lack of a precise description of myocardial infarction or its pathogenesis in the red panda, we can only speculate about lesion pathogenesis based on an analogy with the most common type of human myocardial infarction—the anterior myocardial infarct. This subtype results from ischemic events along the anterior interventricular branch.^8^ In animals, this corresponds to the paraconal interventricular branch, where lesions occurred in our case. In humans, myocardial infarction, defined as myocardial cell death attributed to prolonged ischemia, is mostly caused by thrombotic events following atherosclerotic plaque rupture within the coronary vessels (syn. atherothrombotic coronary artery disease),^13^ which was not present in our case. Nevertheless, a rare subtype of myocardial infarction in humans exists, in which myocardial ischemia occurs without any lesions in the coronary arteries, and is termed “myocardial infarction with non-obstructive coronary arteries” (**MINOCA**).^13^ Most of these MINOCA-type myocardial lesions are caused by neoplasms that obstruct the coronary arteries from the outside. There are reports of both a thymoma and a thymic carcinoma causing MINOCA-type myocardial infarction in humans.^1,14^

No evidence was found of any thrombi during the diagnostic workup of our case, and the animal’s coronary arteries were without any significant findings. Thus, we propose that the neoplasm’s mass effect compressed the left coronary artery, leading to the ischemic event(s) responsible for the myocardial infarcts. The infarcts were chronic; hence, we cannot rule out that thrombi might have existed and resolved before examination, although this is considered unlikely. Additionally, cancer in general can facilitate thrombosis through multiple prothrombotic paraneoplastic mechanisms. Beyond mere mass effects, cancer can both directly and indirectly stimulate the clotting system, resulting in a markedly increased risk of thrombosis.^6^ Although different neoplasms vary considerably in their paraneoplastic promotion of a hypercoagulable state, a B2 thymoma and thymic carcinoma have been reported to cause consecutive thromboses in human patients^2,3^; a similar correlation might be plausible in our case.

Intralesional carbon particles and crystals were present within macrophages in the tracheobronchial lymph nodes, as well as the lung. This finding suggests a longstanding case of pulmonary anthracosilicosis with repeated lymphatic drainage, which led to a prominent silicate-induced granulomatous lymphadenitis, as described previously.^12,17^ Based on our overall findings and the intralesional colocalization of aluminum and silicate in particular, we hypothesize that aluminum-silicate complexes acted as the primary trigger for granulomatous inflammation. In contrast to other reports of silicosis in wildlife species, such as badgers and mongooses,^12,17^ the red panda is not a burrowing, soil-dwelling species. As an arboreal herbivorous mammal, which occupies a distinct ecologic niche within its wildlife habitat,^19^ the red panda would therefore not be exposed to aluminum-complexed silicates. Hence, we propose that the most likely source of these complexes in our case was the maintenance and abrasion of concrete surfaces within the artificial environment of the zoo structure in which the animal was housed, possibly in combination with deposition of urban particulates.

The elevated concentrations of creatinine, urea nitrogen, and phosphate indicated renal azotemia that resulted from the moderate interstitial nephritis, which is a common degenerative renal lesion in aged red pandas.^4,10^ Also, bacteria and fungi were detected in all 7 examined organs. The systemic presence of the obligate pathogens *Salmonella* and *Clostridium* spp. might indicate terminal septicemia; however, in the absence of correlating histopathologic alterations, it could also result from postmortem bacterial translocation and growth.

## Supplemental Material

sj-pdf-1-vdi-10.1177_10406387261422673 – Supplemental material for Thymoma with suspected secondary myocardial infarction in a red panda Supplemental material, sj-pdf-1-vdi-10.1177_10406387261422673 for Thymoma with suspected secondary myocardial infarction in a red panda by Susanne Rau, Andreas Bernhard, Regina Scheller and Jan Schinköthe in Journal of Veterinary Diagnostic Investigation
