# Supplementary material for: Thymoma with suspected secondary myocardial infarction in a red panda
Source: J Vet Diagn Invest. 2026 Apr 4:10406387261422673. Online ahead of print. doi: 10.1177/10406387261422673 (PMC13050361; doi:10.1177/10406387261422673)
Supplement: sj-pdf-1-vdi-10.1177_10406387261422673 – Supplemental material for Thymoma with suspected secondary myocardial infarction in a red panda [file sj-pdf-1-vdi-10.1177_10406387261422673.pdf]

Rau S, et al. Thymoma with suspected secondary myocardial infarction in a red panda

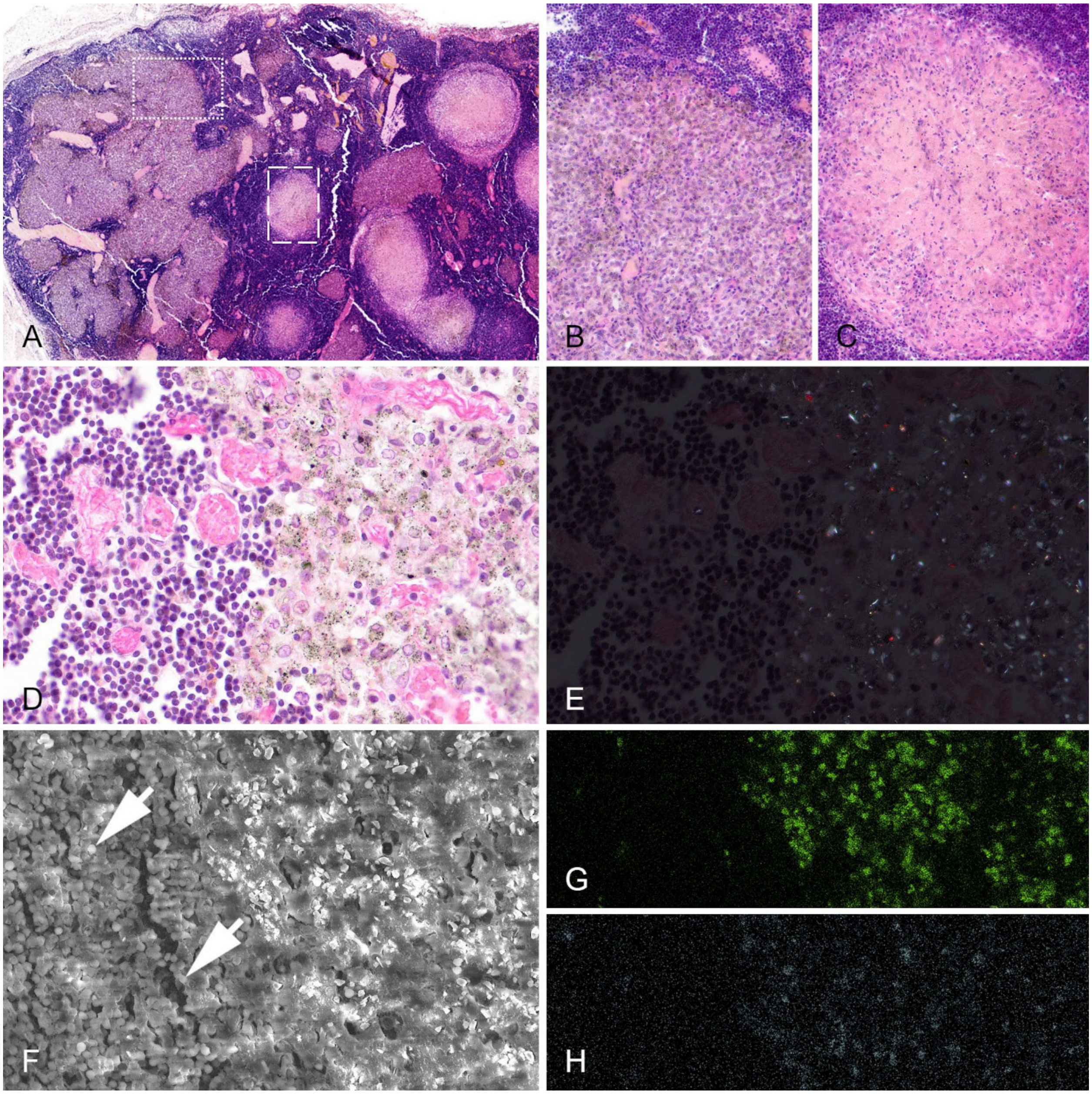

Supplemental Figure 1. Granulomatous lymphadenitis with anthracosilicosis of the tracheobronchial lymph nodes in a red panda. **A.** Lymph node architecture is severely disrupted by granulomas with central areas of caseous necrosis (dashed rectangle). H&E. **B.** Non-caseating

granuloma in the dotted-border rectangle in Suppl. Fig. 1A. H&E. **C.** A granuloma with prominent central caseous necrosis surrounded by epithelioid macrophages in the dashed-border rectangle in Suppl. Fig. 1A. H&E. **D.** Periphery of the granuloma in Suppl. Fig. 1B with epithelioid macrophages containing abundant brown-black carbon particles (anthracosis). H&E. **E.** Widespread accumulation of intralesional birefringent amorphous crystals. Polarized H&E. **F.** Scanning electron microscopy image of a consecutive section with round cells interpreted as lymphocytes (arrows) adjacent to numerous white crystalline structures on the right. **G, H.** Energy-dispersive x-ray spectroscopy mapping reveals abundant silica- (green dots) and aluminum-specific signals (silver dots). Accelerating voltage: 15 keV, collection time: 30 s.
